# A clinically relevant polymorphism in the Na^+^/taurocholate cotransporting polypeptide (NTCP) occurs at a rheostat position

**DOI:** 10.1074/jbc.RA120.014889

**Published:** 2020-12-02

**Authors:** Melissa J. Ruggiero, Shipra Malhotra, Aron W. Fenton, Liskin Swint-Kruse, John Karanicolas, Bruno Hagenbuch

**Affiliations:** 1 Department of Pharmacology, Toxicology and Therapeutics, The University of Kansas Medical Center, Kansas City, Kansas, USA; 2 Program in Molecular Therapeutics, Fox Chase Cancer Center, Philadelphia, Pennsylvania, USA; 3 Center for Computational Biology, University of Kansas, Lawrence, Kansas, USA; 4 Department of Biochemistry and Molecular Biology, The University of Kansas Medical Center, Kansas City, Kansas, USA

**Keywords:** bile acid transport, drug transport, single nucleotide polymorphism, hepatocyte, liver, protein stability, ASBTs, apical sodium-dependent bile acid transporters, HEK293, human embryonic kidney 293 cells, NaCl, sodium chloride, NTCP, Na^+/^TCA cotransporting polypeptide, PBS, phosphate-buffered saline, PDB, Protein Data Bank, SLC10A, solute carrier family 10A, TBS, Tris-buffered saline, TCA, taurocholate, TM, transmembrane, WT, wildtype

## Abstract

Conventionally, most amino acid substitutions at “important” protein positions are expected to abolish function. However, in several soluble-globular proteins, we identified a class of nonconserved positions for which various substitutions produced progressive functional changes; we consider these evolutionary “rheostats”. Here, we report a strong rheostat position in the integral membrane protein, Na^+^/taurocholate (TCA) cotransporting polypeptide, at the site of a pharmacologically relevant polymorphism (S267F). Functional studies were performed for all 20 substitutions (S267X) with three substrates (TCA, estrone-3-sulfate, and rosuvastatin). The S267X set showed strong rheostatic effects on overall transport, and individual substitutions showed varied effects on transport kinetics (*K*_m_ and *V*_max_) and substrate specificity. To assess protein stability, we measured surface expression and used the Rosetta software (https://www.rosettacommons.org) suite to model structure and stability changes of S267X. Although buried near the substrate-binding site, S267X substitutions were easily accommodated in the Na^+^/TCA cotransporting polypeptide structure model. Across the modest range of changes, calculated stabilities correlated with surface-expression differences, but neither parameter correlated with altered transport. Thus, substitutions at rheostat position 267 had wide-ranging effects on the phenotype of this integral membrane protein. We further propose that polymorphic positions in other proteins might be locations of rheostat positions.

Amino acid substitutions are commonly used to evaluate which amino acids in a protein contribute to function. Several decades of studies have led to conventional “rules” for mutational outcomes that are now included in many textbooks and are often implicitly or explicitly assumed in the design and interpretation of experimental studies. For instance, at “important” protein positions, only amino acids with biochemical properties similar to the wildtype (WT) are expected to allow function, whereas other amino acid substitutions are expected to abolish function or structure. However, the mutational studies that gave rise to these rules were primarily focused on evolutionarily conserved amino acid positions (1). When we performed substitution studies of less conserved positions, results were seldom consistent with expected outcomes. Instead of an on/off pattern, when nonconserved positions were substituted with a variety of amino acids, each substitution had a different outcome. The fact that one position could be substituted to access a continuum of functional outcomes is analogous to an electronic dimmer switch; therefore, these positions have been labeled as “rheostat” positions (2, 3, 4).

To date, biochemical studies of rheostat positions have been limited to a few positions within a few proteins. As of yet, there are insufficient data to demonstrate how widespread such positions are in the protein universe or their general properties. As part of our efforts to expand general knowledge of rheostat positions, we chose the integral membrane transport protein human Na^+^/taurocholate (TCA) cotransporting polypeptide (NTCP) as a model system, which allowed us to address three specific areas of interest.

First, we were curious whether rheostat positions were limited to the soluble-globular class of proteins in which they were discovered (3), or if they also exist in transmembrane (TM) proteins. Because soluble and integral membrane proteins evolved under different chemical environments, the properties of one class are not always transferable to the other. In the context of rheostat positions, it helps to know in which types of proteins to expect them. Predictions about substitutions at rheostat positions require different algorithms than predictions at positions that follow textbook substitution rules (5, 6).

Second, if rheostat positions do exist in integral membrane proteins, we wondered whether the functional outcomes arising from various substitutions were dependent on the substrate being transported. That is, we wished to explore the effects of substitutions at rheostat positions on substrate specificity. Although our prior work suggested such substitutions could have complex effects on specificity (7), that work was carried out with non-natural proteins and ligands. The present study, using a natural protein known to transport multiple different substrates, provided opportunity to further document this complex substitution outcome. Amino acid changes that alter substrate specificity are key to the evolution of functional variation and may thus also give rise to different drug sensitivities.

Third, many of the rheostat positions identified to date are located outside binding sites and do not directly contact ligand or substrate. As such, their molecular mechanisms of action have been difficult to explain (8). Recent studies of a rheostat position in a transcription repressor have indicated that substitutions may alter protein dynamics (9). Thus, the present study provided opportunity to relate the continuum of functional outcomes of the rheostat variants to the complex conformational changes experienced by an integral membrane protein during transport.

Several features of NTCP facilitated the studies listed previously: This protein is expressed at the basolateral membrane of human hepatocytes where it plays an important role in the enterohepatic circulation of bile acids (10). In addition to conjugated bile acids such as TCA, NTCP mediates the uptake of other substrates into hepatocytes, including estrone-3-sulfate and several statins such as rosuvastatin (11). Furthermore, several single nucleotide polymorphisms have been reported to alter the transport activity of NTCP (12), which we reasoned might enable the identification of rheostat positions from the 349 positions that comprise this protein.

From various analyses, we identified position 267 as a potential rheostat position in NTCP. Next, we assessed the function of WT NTCP and all 19 amino acid substitutions at position 267 with cellular uptake studies. We also determined whether substitution outcomes were substrate dependent by measuring transport of TCA, estrone-3-sulfate, and rosuvastatin. Additional experiments differentiated the effects of substitutions on protein surface expression and transport kinetics. Finally, we used homology modeling of the available “inward-open” and “outward-open” conformations and energetic calculations to explore the “rheostatic” relationship between protein stability and surface expression.

## Results

Because generalizable features for identifying rheostat positions have not yet been validated, we first faced the challenge of identifying a likely candidate among the 349 amino acid positions of NTCP. Thus, we combined two types of information—functional insights and sequence analysis—to identify a likely rheostat position for the present study.

The functional information derived from knowledge of NTCP polymorphisms with clinical consequences. The most frequent and best characterized of these is NTCP∗2, which leads to the missense amino acid substitution S267F and has an allele frequency of 7.5% in Chinese Americans. Previously published data for S267F indicated reduced transport of TCA, WT-like transport of estrone-3-sulfate, and increased transport of rosuvastatin *in vitro* (12, 13, 14). Clinically, this mutation resulted in severe hypercholanemia with total serum bile acid levels of about 15- to 70-fold above normal in homozygous pediatric patients (15, 16); some of the patients also had elevated liver enzymes, jaundice, and gallstones (16). In homozygous adult patients, NTCP∗2 resulted in total serum bile acid levels two- to fivefold above normal (17).

The other prevalent polymorphism is NTCP∗3, which is found in 5.5% of African Americans and results in the amino acid substitution I223T. However, the resulting protein expression at the plasma membrane was significantly reduced compared with WT NTCP (12). Inadequate plasma membrane expression would make functional studies problematic; thus, this single nucleotide polymorphism was not further explored in the current work.

Polymorphic positions are, by definition, nonconserved. Likewise, the previously identified rheostat positions in the soluble LacI/GalR homologs were also nonconserved and had moderate to high phylogeny scores (3, 7). Thus, to further strengthen our reasoning that NTCP position 267 should be explored as a potential rheostat position before embarking on experiments, we assessed these evolutionary properties. Position 267 was previously reported to be highly conserved among many different animals, including primates, rodents, dogs, cats, horses, chicken, several fish, and marine chordate (15). However, when we expanded the sequence alignment to include 1561 homologs of the solute carrier family 10A (SLC10A) (11) representing all kingdoms of life (see NTCP Sequence Alignment File in the Supporting Information), position 267 had a sequence entropy of 1.54. This intermediate conservation score (overall range of 0.0–2.8) suggested that position 267 could tolerate multiple substitutions without catastrophic outcomes, which is requisite for most amino acid substitutions at rheostat positions.

When we further analyzed the expanded sequence alignment with ConSurf (https://consurf.tau.ac.il) (18), position 267 had a score of 8 (on a scale of 1–9), indicating a pattern of change that highly correlated with the branching of the SLC10A phylogenetic tree. This characteristic has been hypothesized to be indicative of positions important for evolving functional variation, (*e.g.* [19, 20]), which may be a key biological role of rheostat positions (3, 8).

### Cellular substrate transport by S267 variants

**Figure 1:**
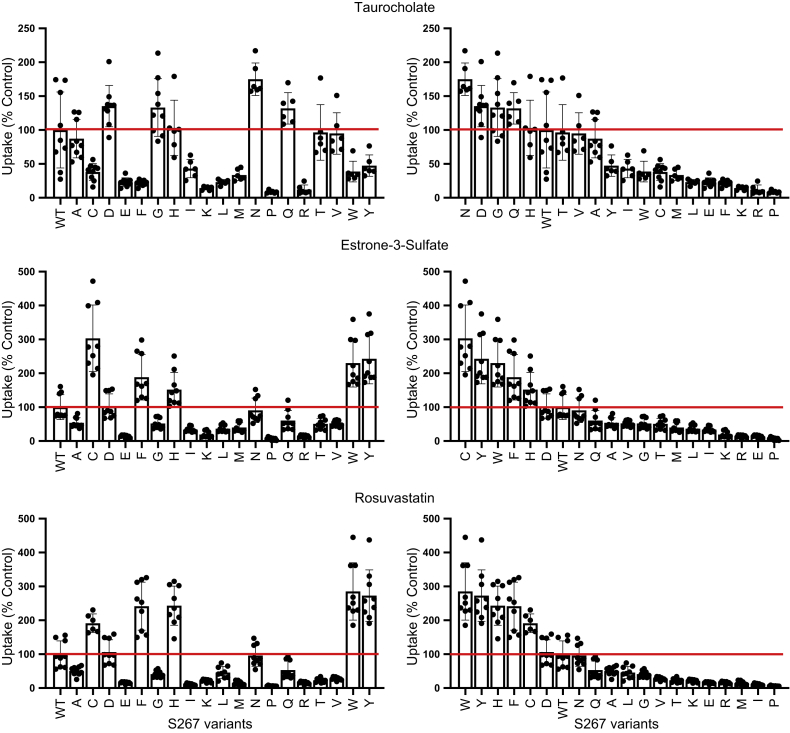
**Substrate uptake by WT NTCP and S267 variants.** Uptake of ^3^[H]taurocholate (30 nM), ^3^[H]estrone-3-sulfate (5.8 nM), and ^3^[H]rosuvastatin (50 nM) was measured for 5 min at 37 °C, 48 h after transfection of wildtype NTCP, its S267 variants, and empty vector into human embryonic kidney 293 cells. Net uptake was obtained by subtracting the uptake of cells transfected with empty vector from uptake of NTCP-expressing cells. The left-hand side shows the results ordered alphabetically based on the amino acid replacement, and the right-hand side shows the substitutions ordered from highest to lowest transport activity. Results were calculated as percent of WT NTCP. Individual data points as well as the mean ± SD are reported from n = 3 biological replicates (each with two to three technical replicates) for all but rosuvastatin uptake by S267C for which n = 2 biological replicates are shown. Horizontal lines to aid visual inspection correspond to WT values, which were set to 100%; results of statistical analyses are shown in Table S1. NTCP, Na^+^/taurocholate cotransporting polypeptide; WT, wildtype.

To experimentally ascertain whether position 267 was a rheostat position, we replaced serine with all other n19 amino acids and measured uptake of TCA (Fig. 1, top), estrone-3-sulfate (Fig. 1, middle), and rosuvastatin (Fig. 1, bottom) for each substitution. On the left side of Figure 1, uptake is shown with the amino acids in alphabetical order, with WT at far left. The right panels show results ordered from highest to lowest transport, with WT placed within the series.

Notably, for all three substrates, some variants transported substrates better than WT, whereas others had diminished transport. The range of observed changes spanned several orders of magnitude. Thus, position 267 exhibited definitive rheostatic substitution behavior. Furthermore, the rank order of the amino acid substitutions differed among the three substrates. This is further discussed later, but here, we particularly note that the NTCP∗2 polymorphism, S267F, showed a significant decrease in TCA transport, a 2.0-fold increase for estrone-3-sulfate transport, and 2.5-fold increase for rosuvastatin transport. Previous reports showed decreased TCA transport, estrone-3-sulfate levels similar to WT, and increased rosuvastatin transport (12, 13, 14). The discrepancy for estrone-3-sulfate could arise from the slightly different uptake conditions, including substrate concentrations, incubation time, and/or different cell lines used in the two studies.

### Dissecting the composite cellular outcomes of S267 variants

Protein substitutions can alter substrate transport kinetics, substrate specificity, protein stability, and/or intracellular trafficking to the outer membrane. The cellular uptake assay is sensitive to changes in any of these parameters. Thus, we devised experiments to dissect the functional and structural contributions.

**Figure 2:**
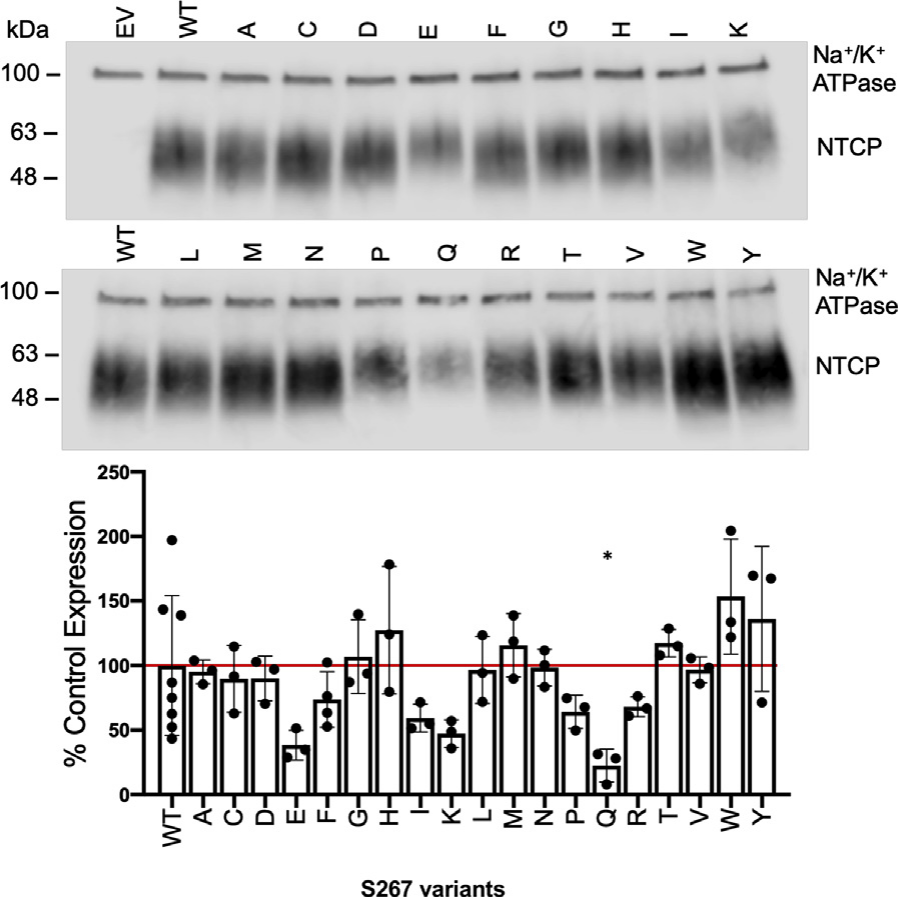
**Surface expression of WT NTCP and S267 variants.***A*, representative Western blot of surface-expressed WT NTCP and its S267 variants in transiently transfected human embryonic kidney 293 cells. EV, WT, and S267 variant proteins were separated on a 4% to 20% gel and then transferred to a nitrocellulose membrane. Blots were probed with a combination of Na^+^/K^+^-ATPase (loading control at 100 kDa) and tetra-His antibodies (recognizes the His-tagged transporter). *B*, quantification of S267 variants relative to WT NTCP. Expression was quantified using Image Studio Lite, and the bars represent the mean ± SD of three independent experiments; an asterisk indicates a *p* < 0.05 level of significant difference from WT NTCP. The horizontal line indicates WT control, which was set to 100%. EV, empty vector; NTCP, Na^+^/taurocholate cotransporting polypeptide; WT, wildtype.

To assess the combined effects of trafficking and stability, we quantified differences in surface expression of NTCP variants using surface biotinylation experiments followed by Western blotting (Fig. 2*A*). When normalized for the loading control Na^+^/K^+^ATPase, the expression levels varied between 22.5% for S267Q and 153% for S267W, corresponding to an overall variation of about sevenfold. The expression of most of the other variants was similar to WT (Fig. 2*B*). Thus, NTCP appeared to accommodate different amino acid side chains at position 267 without significantly disrupting the overall structure or trafficking to the cell surface.

**Figure 3:**
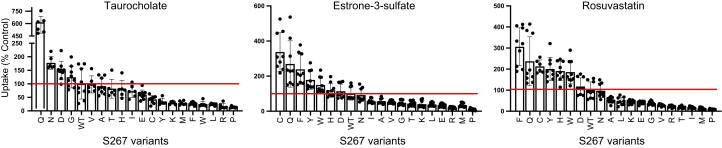
**Initial substrate uptake normalized for surface expression.** Uptake results (Fig. 1) were corrected for the surface expression (Fig. 2*B*) and are presented with the substitutions rank ordered from largest to smallest for each substrate. Horizontal lines indicate wildtype control, which was set to 100%. Error bars represents propagated SD; results of statistical analyses are shown in Table S2.

**Figure 4:**
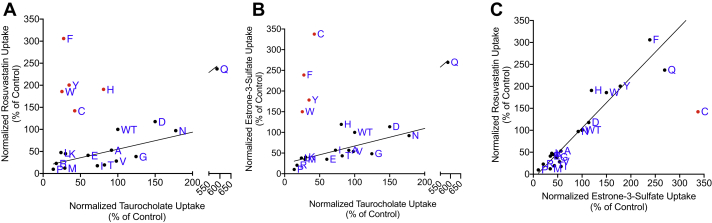
**Comparison of normalized substrate uptake among the three different substrates.** Normalized uptake values from Figure 3 are plotted against each other. Individual points are labeled with letters to indicate their amino acid replacements; wildtype is indicated as WT. *A*, ^3^[H]taurocholate *versus*^3^[H]estrone-3-sulfate, *B*, taurocholate *versus*^3^[H]rosuvastatin, and *C*, E3S *versus*^3^[H]rosuvastatin. On each panel, several substitutions (*red dots*) were outliers as compared with the others (*black dots*), which is a hallmark of altered substrate specificity. For the nonoutlier substitutions, the Pearson coefficient, which is a measure of the linear correlation, was >0.91 (*p* < 0.0001) for all three substrate comparisons. The Spearman coefficient, which correlates rank order, also showed a strong correlation with values of 0.7 to 0.87 (*p* < 0.0001) (Table S3). For the correlated substitutions, the slopes of the three trend lines were *A*, 0.42 (95% confidence intervals of 0.32–0.52), *B*, 0.39 (0.28–0.49), and *C*, 1.15 (0.96–1.34), respectively. Slopes that differ significantly from 1 are another hallmark of altered specificity (7).

Next, initial uptake experiments from Figure 1 were corrected for the surface expression, and results are shown in Figure 3. The overall rheostat-like behavior remained, showing that substitutions altered transport function. The rank orders of S267 substitutions were further analyzed using correlation plots to illustrate the effects on substrate specificity (Fig. 4). Inspection of these plots showed that striking specificity differences were evident for four amino acids (C, F, W, and Y) in the comparison of TCA to estrone-3-sulfate, for five amino acids (C, F, H, W, and Y) for TCA to rosuvastatin, and for one amino acid (C) for estrone-3-sulfate to rosuvastatin. The differential effects of these substitutions—including the clinically relevant polymorphism F—for transporting alternative substrates are a hallmark of altered substrate specificity (7).

Of note, correlation with TCA uptake showed similar outliers for estrone-3-sulfate and rosuvastatin, whereas only one outlier was identified when comparing estrone-3-sulfate and rosuvastatin. This suggests that estrone-3-sulfate and rosuvastatin, with the highest Pearson and Spearman values (Fig. 4*C* and Table S1), share similar modes of interaction with position 267.

When these outlier variants were excluded, the remaining variants showed significant correlation (Fig. 4 and Table S3), illustrating that the amino acid substitutions had the same direction of change for the alternative substrates. For rosuvastatin and estrone-3-sulfate, the slope of the line was near 1. However, for the other two correlations, the slopes of the lines were less than 1, which is another sign of altered specificity for TCA by these substitutions ([7] and references therein).

**Figure 5:**
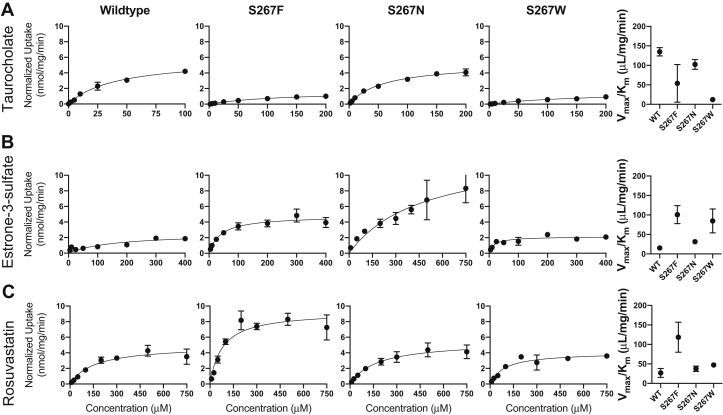
**Kinetics of substrate transport mediated by WT Na**^**+**^**/taurocholate (TCA) cotransporting polypeptide and selected variants.** Kinetics of *A*, TCA, *B*, estrone-3-sulfate, and *C*, rosuvastatin uptake by wildtype Na^+^/TCA cotransporting polypeptide (first column), S267F (second column), S267N (third column), S267W (fourth column), and transport capacity (fifth column). Uptake of increasing concentrations of each substrate was measured under initial linear rate conditions in human embryonic kidney 293 cells 48 h after transfection. Results shown are the mean ± SD of a representative experiment completed with triplicate technical samples. The curves are best fits of the mean values using the Michaelis–Menten equation in GraphPad Prism 8. The results listed in Table 1 report the average and SD of at least three independent experiments, each comprising two to three technical replicates. WT, wildtype.

Based on the results presented in Figure 3, we performed a full kinetic analysis for select variants: WT, S267F (the NTCP∗2 polymorphism), S267N, and S267W. Asparagine at position 267 was chosen because this substitution resulted in similar uptake as WT for estrone-3-sulfate and rosuvastatin but higher uptake for TCA. In contrast, the tryptophan substitution was chosen because it resulted in lower uptake for TCA but higher uptake for estrone-3-sulfate and rosuvastatin. For these four proteins, concentration dependent uptake was assessed under initial linear rate conditions using transiently transfected human embryonic kidney 293 (HEK293) cells. After normalizing for surface expression, we analyzed the results using the Michaelis–Menten equation and calculated *K*_m_ and *V*_max_ values (Fig. 5 and Table 1). Both the *K*_m_ and *V*_max_ were frequently altered, indicating altered substrate affinity and transporter turnover.

**Table 1:** Kinetic values for substrate uptake mediated by WT NTCP and selected variants

Substrate	NTCP	S267F	S267N	S267W
TCA				
*K*_m_ (μM)	42 ± 6.6	73 ± 56	59 ± 18	153 ± 50
*V*_max_ (nmol/mg/min)	5.7 ± 0.5	1.9 ± 0.6	5.9 ± 1.8	1.7 ± 0.1
*V*_max_/*K*_m_ (μl/mg/min)	134 ± 24	26 ± 22	102 ± 44	11 ± 3.7
Estrone-3-sulfate				
*K*_m_ (μM)	155 ± 18	42 ± 5.7	420 ± 173	21 ± 6.7
*V*_max_ (nmol/mg/min)	2.3 ± 0.2	4.2 ± 0.6	13 ± 4.7	1.7 ± 0.5
*V*_max_/*K*_m_ (μl/mg/min)	15 ± 2.3	99 ± 19	31 ± 17	81 ± 33
Rosuvastatin				
*K*_m_ (μM)	183 ± 51	73 ± 21	171 ± 26	88 ± 12
*V*_max_ (nmol/mg/min)	4.5 ± 1.0	8.2 ± 1.7	6.3 ± 0.9	4.1 ± 0.4
*V*_max_/*K*_m_ (μl/mg/min)	25 ± 8.9	112 ± 39	37 ± 7.6	47 ± 7.4

NTCP, Na^+^/taurocholate cotransporting polypeptide; TCA, taurocholate; WT, wildtype.

Uptake of increasing concentrations of TCA, estrone-3-sulfate, and rosuvastatin by human embryonic kidney 293 cells transiently transfected with either WT NTCP or variants S267F, S267N, and S267W was measured at 37 °C under initial linear rate conditions. Net uptake was calculated by subtracting the transport from identical experiments using sodium-free buffer and was normalized for surface expression. Kinetic parameters, *K*_m_ and *V*_max_, were determined by fitting the data to the Michaelis–Menten equation using GraphPad Prism 8. Transport capacity was determined by dividing the *V*_*max*_ by the *K*_*m*_. The parameter averages and standard deviation presented were calculated from at least three independent experiments, each comprising three technical replicates.

To summarize these data, we calculated the capacity of each variant to transport the various substrates (*V*_max_/*K*_m_) (Table 1). For the most part, the capacity of the variants chosen agreed with the rank order shown in Figure 3. Of the four variants, WT NTCP had the highest capacity (134 ± 14 μl/mg/min) for TCA (Fig. 5*A* and Table 1) but the lowest capacity for the other two substrates: 15 ± 1.3 μl/mg/min for estrone-3-sulfate and 24 ± 5.1 μl/mg/min for rosuvastatin. The lowest capacity for TCA (11 ± 2.1 μl/mg/min) was determined to be for S267W (Fig. 5*A* and Table 1), in agreement with the single time point single concentration results (Fig. 3*A*). For estrone-3-sulfate and rosuvastatin (Fig. 5, *B*–*C* and Table 1), S267F showed the highest capacity with 99 ± 11 μl/mg/min and 112 ± 23 μl/mg/min, respectively, which confirms the results presented in Figure 3, *B*–*C*. In summary, amino acid substitutions at rheostat position 267 differentially altered both kinetic parameters for substrate transport.

### Homology modeling of human NTCP structure

**Figure 6:**
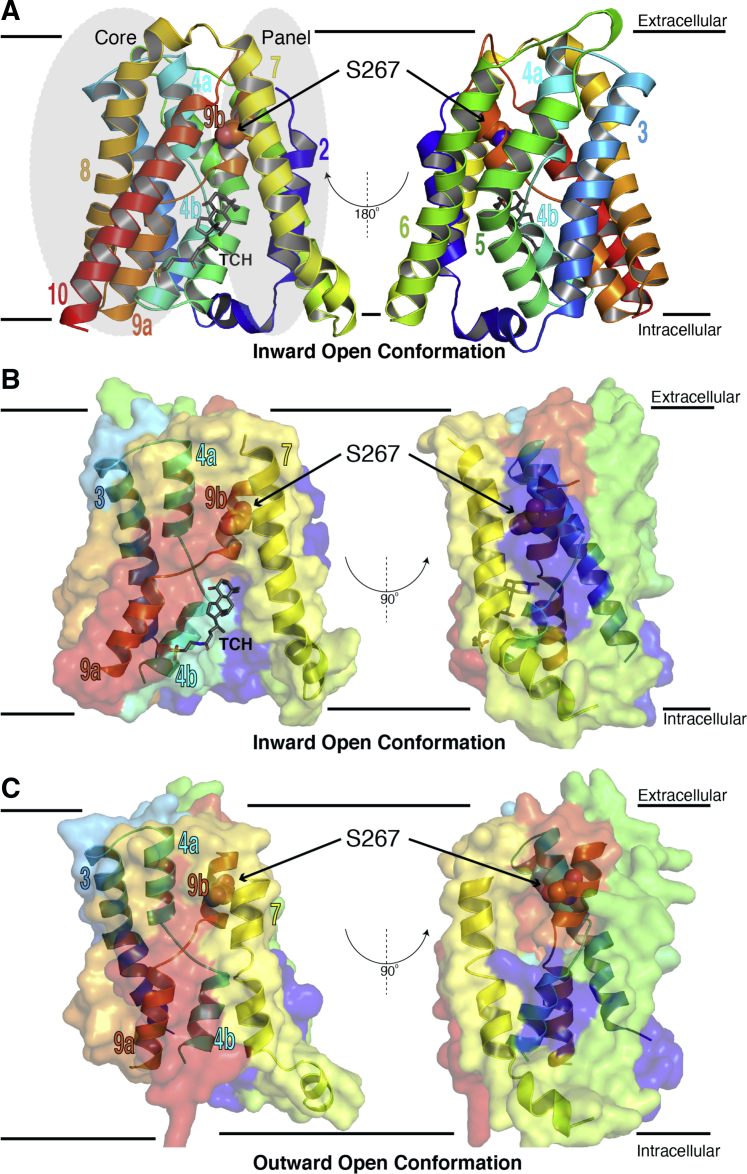
**Comparative models of human NTCP.***A*, homology model of human NTCP in the inward-open conformation, built using apical sodium-dependent bile acid transporter from *Neisseria meningitidis* as a structural template. The structure comprises nine TM helices (denoted TMs 2, 3, 4a, 4b, 5–8, 9a, 9b, and 10). Position 267 is located on TM 9b. The inward-open conformation has a large crevice at the intracellular side of membrane that is formed between the core and panel domains. Taurocholate (TCH), in gray sticks, is included in this perspective by superimposition from the template to show the interaction of a substrate in the inward-open conformation. *B*, the inward-open model is shown in cross section, to highlight the arrangement of helices in this conformation. *C*, homology model of human NTCP in the outward-open conformation, built using apical sodium-dependent bile acid transporter from *Yersinia frederiksenii* as a structural template. Relative to the inward-open conformation, the substrate-binding pocket has closed in this conformation, because of a concerted movement of TM helices 3, 7, 4a, 9b, 4b, and 9a. NTCP, Na^+^/taurocholate cotransporting polypeptide; TM, transmembrane.

Both modeling and experimental data suggest that human NTCP has nine TM helices (21, 22) with an extracellular glycosylated amino terminus (23). These helices are arranged into “core” (TM 3, 4, 5, 8, 9, and 10) and “panel” domains (TM 2, 6, and 7) that flank the substrate-binding intracellular crevice (*e.g.,* Fig. 6). Although no experimental structure is available for human NTCP, structural information could be derived from homology studies. Homologs in the SLC10A family exhibit 9.5% to 99.0% sequence identity and are present in most kingdoms of life (Supporting Information). Bacterial apical sodium-dependent bile acid transporters (ASBTs) are the best-structurally characterized; structures are available for *Yersinia frederiksenii* ASBT (ASBT_Yf_, 26% sequence identity to NTCP) (24) and *Neisseria meningitidis* (ASBT_Nm_, 25% sequence identity to NTCP) (22). The pairwise sequence alignments of both relative to NTCP are presented as Fig. S1.

In ASBT_Yf_, transport of bile acids and other substrates appears to be accomplished by a transition between inward-open and outward-open conformations. More specifically, this is accomplished *via* a rigid body motion of the core domain (24) that allows alternating exposure of ligand-binding sites to the intracellular or the extracellular space. Both ASBT and NTCP cotransport sodium ions along with bile salts. In the crystal structure of ASBT_Nm_, two Na^+^-binding sites have been identified at the junction of core and panel domains (24).

We used the inward-open and outward-open crystal structures of two bacterial ASBTs as templates for comparative modeling of human NTCP. We then applied all-atom structural refinement to the homology models to generate the lowest energy inward-open and outward-open homology models for WT human NTCP. In agreement with the bacterial structures, a well-defined pocket was present at the junction of the core and panel domains (Fig. 6, *A*–*B*), in which TCA could bind before being transported. Comparison of the inward- and outward-open models suggest that conformational changes in TM domains 3, 4a, 4b, 7, 9a, and 9b led to closing of that pocket and an opening of a pocket where TCA binds before being released intracellularly (Fig. 6*C*). Position S267 was located on TM9b, near the substrate-binding cavity in both the inward- and outward-open conformations, and substitutions therefore may directly influence substrate transport.

### Evaluating stability changes arising from substitutions at position S267

**Figure 7:**
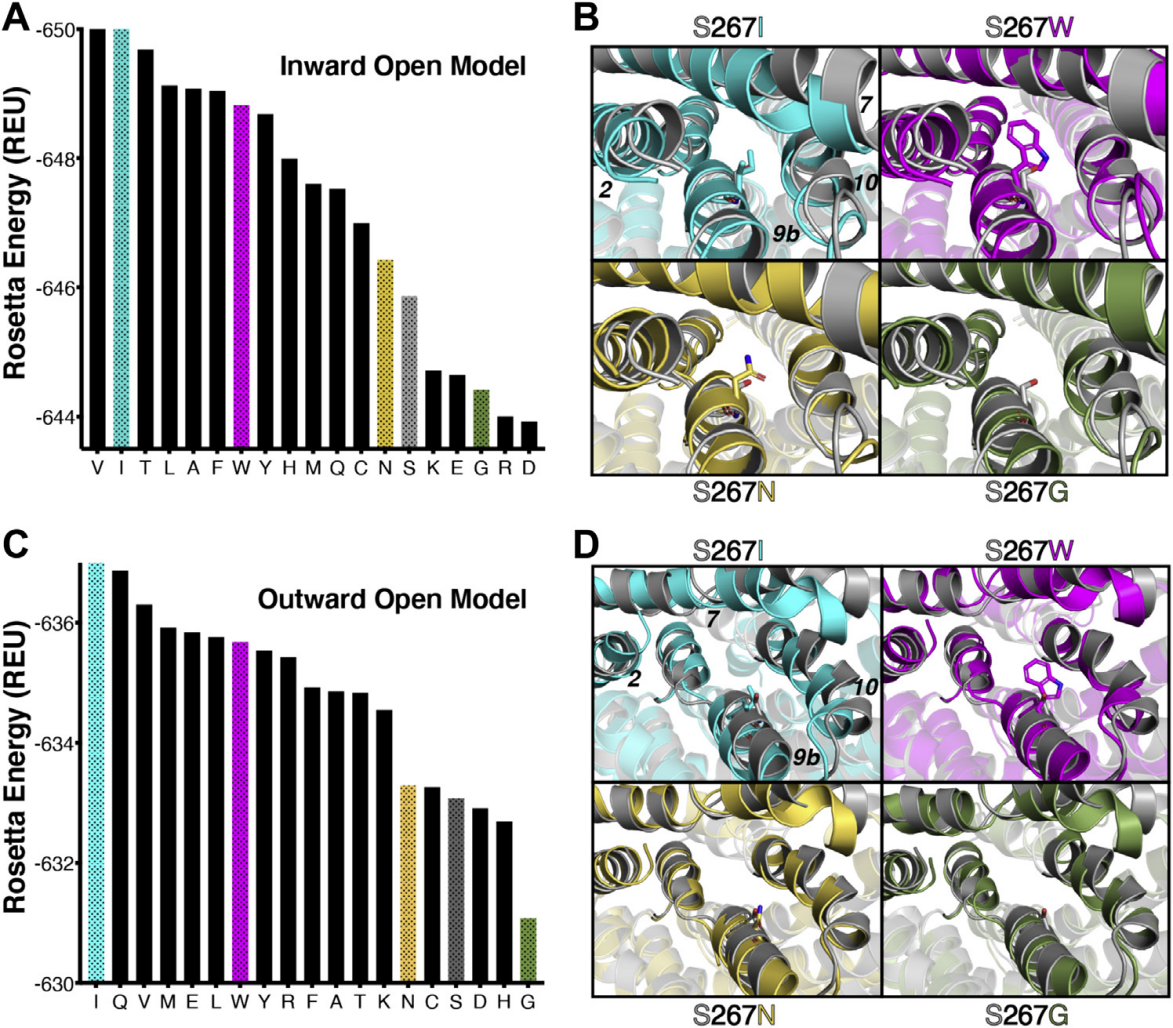
**Predicted stability differences associated with sequence variation at the S267 position.***A*, Rosetta energies for 19 sequence variants at the 267 position, using the inward-open model. Proline is not shown, because the energy associated with this residue is very unfavorable (off the scale); this is consistent with experimental results, in which proline showed the least amount of transport and somewhat diminished surface expression (Figure 1, Figure 2, Figure 3). *B*, structural details from the models underlying these energy differences. Four different sequence variants are compared with the wildtype S267; in each case, the conformation of TM helices 2 and 7 respond to changes in the amino acid at position 267, which is located on TM helix 9b. *C*, Rosetta energies using the outward-open model. Proline is again not shown, because the energy associated with this residue is very unfavorable (off the scale). *D*, structural details from the outward-open models. In this conformation, the position of TM helix 10 responds to changes in the amino acid at position 267. REU, Rosetta energy unit; TM, transmembrane.

Next, we modeled all 19 amino acid substitutions at position 267 of the WT homology models and assessed the predicted stability changes on the inward- and outward-open conformations. Starting with the inward-open conformation, we found that most substitutions were predicted to be stabilizing relative to the WT control (serine at position 267): 13 of the 19 potential substitutions yield energies more favorable (more negative) than serine (Fig. 7*A*). This observation was striking because it is in stark contrast with typical results from such computation predictions, which have long found that the WT amino acid tends to score more favorably than any other substitution (25).

To explore the structural basis for the observed energetics in this modeling experiment, we selected two substitutions that were stabilizing (S267I and S267W), one with little effect on stability (S267N) and one predicted to be slightly destabilizing (S267G). Inspection of the representative conformations for each of these variants revealed slight changes in the local environment; in particular, TM helices 2 and 7—which face the side chain presented at position 267—respond in a slightly different manner to each variant (Fig. 7*B*).

In our model of the outward-open conformation, the WT S267 was more solvent exposed than in the inward-open conformation, with a solvent-accessible surface area of 17.76 Å^2^ as compared with 4.26 Å^2^. Remarkably though, the same unusual behavior emerged when probing stability differences in the outward-open conformation. Many substitutions were predicted to be more stable than the WT serine (Fig. 7*C*), and again the notable tolerance for alternate amino acids at position 267 could be rationalized by malleability of the local structure, this time the nearby TM helix 10 (Fig. 7*D*). In both cases, the small rearrangement of these helices understates the dramatic differences in side chain conformations needed to accommodate these alternatively packed arrangements (Fig. S2).

### Correlation of structure models and experimental data

We next compared the computational stabilities of each S267 variant to the experimental data. The most direct comparison should be to the NTCP surface expression, which would be decreased or increased by altered protein stability. To that end, we examined the effect between cellular surface expression levels and the calculated energies using the inward-open NTCP model (Fig. S3*A*), the outward-open model (Fig. S3*B*), and the difference between the energies of the inward-open and outward-open models. We anticipated that if the protein resides primarily in one conformation or the other, its surface expression may correlate with the calculated energies for that conformation; however, we find no statistically significant correlation between surface expression and the energies of the models in either conformation (Table S3).

**Figure 8:**
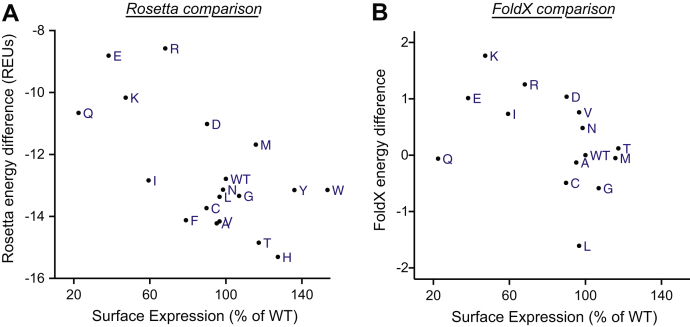
Correlation of surface expression levels with calculated stability differences for inward-open model minus outward-open model. The percent surface expression (Fig. 3) of each variant is plotted against results from structure-based modeling of each variant. Individual points are labeled with letters to indicate their amino acid replacements; wildtype is indicated as WT. Proline is excluded because the stability difference for this helix-breaking residue cannot be reliably estimated using these methods. *A*, energies calculated using Rosetta. *B*, energies calculated using FoldX. Aromatic residues (F/H/W/Y) were excluded because FoldX does not model backbone flexibility, making it unable to accommodate these large side chains. Note that these aromatic residues were the variants that dramatically altered substrate specificity (outliers in Fig. 4). REUs, Rosetta energy units.

Instead, we observed a correlation between surface expression and the difference in energy between the two conformations (Fig. 8*A*) (Pearson and Spearman coefficients were −0.64 and −0.52, respectively; both correlation coefficients were nonzero with *p* < 0.05; Table S3). To rule out any possibility that the dramatic relationship observed between surface expression and the difference in energy between the two conformations was due to some quirk of the Rosetta energy function, we carried out the same calculation using the FoldX package (26). Unsurprisingly, the calculated energies were correlated with those from Rosetta (Fig. S4; *p* < 0.03). We again observed a correlation to the observed surface expression, albeit is not to a statistically significant degree (Fig. 8*B*; *p* < 0.10). We attribute the slightly stronger correlation from Rosetta's calculated energies to the fact that this protocol sought to capture slight backbone rearrangements in response to each mutation, whereas FoldX energies did not include backbone flexibility.

There are several potential explanations for why sequence variants with more favorable energies in the inward-open model relative to the outward-open model (*i.e.*, the difference between the states) yielded higher surface expression. A direct physical interpretation may be that the inward-open conformation promotes surface expression, whereas a stable outward-open conformation serves as a trap that disfavors membrane insertion and/or correct folding required for presentation on the cell surface. In such a model, the outward-open conformation is critical for transport, but overstabilization of this conformation would prove deleterious. As an alternate explanation, however, we note that the Rosetta energy function is parameterized primarily for study of soluble proteins. As a result, energy differences between sequence variants are computed relative to a reference state that may not be appropriate for membrane proteins (the unfolded protein in a soluble context). Because of this, the outward-open conformation (in which position 267 is more solvent exposed) may simply be serving as an alternate (more appropriate) reference state, implying that calculated stability of the inward-open conformation is the primary determinant of surface expression, once energies have been more appropriately normalized.

For completeness, we in addition explored whether there was a relationship between the Rosetta-calculated energies and substrate uptake; we did not expect the Rosetta energies to be predictive, given that the balance of multiple conformational states is likely critical for effective transport. This expectation proved to be the case, as we did not observe a statistically significant correlation between any of the energies with update of any substrate (Fig. S5).

Overall, the strong correlation between surface expression and these computed energy differences supports an underlying structural rationale that diverse amino acids can be accommodated at position 267 in a manner that altered—but did not disrupt—protein activity in a rheostatic manner. Because stability did not appear to be greatly altered, we hypothesize that the experimentally observed changes in transport for each substitution arose from altered dynamics or from altered energies of other conformations that surely exist in the NTCP ensemble.

## Discussion

Rheostat positions were first described in soluble-globular proteins (2, 3) and have been predicted in a wide variety of proteins (6). However, to our knowledge, no such positions have been biochemically/experimentally identified in TM proteins. In the present study, we confirmed that rheostat positions can occur in TM transport proteins such as NTCP. Indeed, the natural polymorphic position, S267, behaved as a rheostat position for all three substrates tested: TCA, estrone-3-sulfate, and rosuvastatin (Fig. 1).

We were further able to parse contributions to NTCP cellular uptake into various biochemical processes, including transport, ligand specificity, membrane localization, and protein stability. Our biochemical approach provided an advantage over the deep mutational scanning studies (27, 28) that have become increasingly popular in recent years. Although the latter experiments substitute large protein regions with all amino acids at each position, they rely on phenotypic competitions that are highly sensitive to environmental conditions/biological thresholds and are the composite of many functional parameters. Furthermore, data interpretation is based on the assumption (and/or requires extensive validation) that allele frequency within a library represents the degree of protein function. Biochemical assays, such as those reported here for NTCP position 267, directly report high-resolution information about the protein's activity that can be resolved into multiple functional and structural components.

Here, the overall phenotype of each substitution at position 267 was primarily dominated by changes in altered transport kinetics. Full kinetic analyses with selected variants demonstrated that both *K*_m_ and *V*_max_ were differentially affected, along with substrate specificity. Nonetheless, both modeling results and experimental data indicated subtle changes in stability that were distinct from (not correlated with) changes in transport. Thus, changes at one rheostat position altered multiple functional and structural parameters, revealing a complex interplay that must be resolved to advance predictive pharmacogenomics. Because we also observed complex outcomes—affecting multiple functional parameters—arising from substitutions at rheostat positions in human liver pyruvate kinase, this complexity is likely common in a variety of proteins (4).

Of the affected functional parameters in NTCP, we were particularly intrigued by the altered substrate specificity that was striking for a subset of the amino acid substitutions at position 267 (changed rank order in Fig. 3 and correlation outliers in Fig. 4). Historically, altered substrate specificity has been defined from the perspective of the proteins, as changes in either the rank order of preferred substrate and/or changes in the fold change of transport (7, 29). The results shown here—from the perspective of the substrate—provide an orthogonal view of specificity.

The substrate-dependent effects became even more apparent when we compared the kinetics for selected variants (Fig. 5 and Table 1). The substitution-dependent effects on substrate specificity could arise if the translocation pathway or binding pocket for TCA differed from that of the other two substrates. Distinct binding pockets within the translocation pathway were recently demonstrated for three substrates of the organic cation transporter 1 (30). Different binding pockets or translocation pathways may be a common feature of multispecific drug transporters. Alternatively, substrate-dependent substitution outcomes might arise if the different substrate/substitution combinations had different effects on the NTCP conformational and equilibrium dynamics, similar to the types of changes that arise from amino acid substitutions in β-lactamase (31).

Indeed, substrate transport by solute carriers like NTCP is a dynamic process generally described by the alternating-access model. As such, NTCP is an intrinsically flexible protein that undergoes complex and hierarchical conformational changes while carrying out its biological function of transporting substrates into hepatocytes. In addition to the outward- and inward-open conformations considered here, NTCP must have multiple intermediate states. The stabilities and/or equilibrium dynamics for each of the conformations could be differentially affected by single amino acid substitutions, giving rise to the intermediate functional outcomes observed. Conformational changes are also modulated by interactions with substrates and sodium ions. Thus, an accurate evaluation of structural characteristics of intermediate conformations along the entire conformational transition pathway will be necessary to understand rheostatic substitution behavior. This remains a challenging task for both experimental and theoretical approaches.

When the rheostatic outcomes from the cellular uptake assays were further investigated, strong rheostatic effects were observed for transport, and the effects on stability of the inward-open conformation (in which position 267 was mostly buried) were slightly rheostatic. Furthermore, computational modeling and stability calculations were in agreement with the experimental measures of protein surface expression. This indicates that modeling merits further exploration for identifying positions in functionally important regions that tolerate a wide range of substitutions, the hallmark characteristic of a rheostat position.

In conclusion, these combined results showed that NTCP position 267 is a rheostat position. Although individual substitutions had wide-ranging effects on the various aspects of function and stability that give rise to the overall phenotype of cellular transport, much of the change could be attributed to altered transport kinetics. Structurally, position 267 was strikingly tolerant to substitution, despite the fact that it was largely buried in the inward-open conformation. Based on these results, we propose that other polymorphic positions in NTCP and in other proteins might also be locations of rheostat positions (8). Given the complex interplay between substitutions and substrate specificity observed at this NTCP rheostat position, it is imperative to expand recognition and understanding of rheostat positions to advance predictive pharmacogenomics.

## Experimental procedures

### Construction of an NTCP sequence alignment

A detailed description of the NTCP sequence alignment is given in the Supporting Information. The references associated with these methods are included in the Supporting Information. The sequence alignment was analyzed with ConSurf (18) to estimate the variation of each NTCP position during evolution.

### Materials

Radiolabeled [^3^H]-TCA (6.5 Ci/mmol) was purchased from PerkinElmer (Boston, MA, USA). [^3^H]-Estrone-3-sulfate (50 Ci/mmol) and [^3^H]-rosuvastatin (10 Ci/mmol) were from American Radiolabeled Chemicals (St Louis, MO, USA). Taurocholic acid sodium salt (97% pure) and estrone-3-sulfate sodium salt (containing 35% Tris stabilizer) were purchased from Sigma Aldrich (St Louis, MO, USA). Rosuvastatin (98% pure) was purchased from Cayman Chemicals (Ann Arbor, MI, USA).

### Site-directed mutagenesis

Mutagenesis reactions were completed using the QuikChange Lightning Multi Site-Directed Mutagenesis Kit (Agilent, Santa Clara, CA, USA). We used a previously cloned His-tagged human NTCP in the pcDNA5/FRT vector for the mutagenesis template (32). Primers were designed to flank the S267 codon by approximately 20 nucleotides and ordered from Invitrogen (Carlsbad, CA, USA). Mutated complementary DNA was then transformed into One Shot TOP10 cells (Thermo Fisher Scientific, Waltham, MA, USA), and bacterial colonies were randomly selected. The complementary DNA was isolated using QIAGEN (Hilden, Germany) miniprep kits and sequenced (GENEWIZ, South Plainfield, NJ, USA). Constructs containing the appropriate amino acid replacements were transfected into HEK293 cells for functional or expression studies as described later.

### Cell culture

HEK293T/17 cells obtained from American Type Culture Collection, Manassas, VA, USA were plated on poly-d-lysine–coated flat bottom 48- and 6-well TPP cell culture plates at densities of 100,000 and 800,000 cells per well, respectively. Cells were cultured in Dulbecco's modified Eagle's medium (American Type Culture Collection) containing 100 U/ml penicillin, 100 μg/ml streptomycin (Thermo Fisher Scientific), and 10% fetal bovine serum (Hyclone; Thermo Fisher Scientific) for 24 h before transfection. Experiments were then completed 48 h after transfection.

### Transient transfection of HEK293 cells

Empty vector, WT NTCP, and variant NTCP plasmids were transfected into HEK293 cells 24 h after plating. Transfections followed the FuGENE HD protocol obtained from Promega (Madison, WI, USA). All plasmids were transfected in triplicates for functional studies on 48-well plates. For surface biotinylation experiments completed on 6-well plates, single wells were transfected.

### Initial uptake experiments

The uptake procedure has been optimized and is well established in our laboratory for functional studies (32). Uptake buffer (142 mM sodium chloride [NaCl], 5 mM potassium chloride, 1 mM monopotassium phosphate, 1.2 mM magnesium sulfate, 1.5 mM calcium chloride, 5 mM glucose, and 12.5 mM Hepes, pH 7.4) was used for all washes and to prepare uptake solutions. Cells were washed with warm uptake buffer and then incubated at 37 °C with nanomolar concentrations of either [^3^H]-TCA (30 nM), [^3^H]-estrone-3-sulfate (5.8 nM), or [^3^H]-rosuvastatin (50 nM) for 5 min. Uptake was then terminated by washing with ice-cold uptake buffer, and the cells were solubilized in a 1% TX-100 solution in phosphate-buffered saline (PBS). Radioactivity was measured using a liquid scintillation counter, and protein concentrations were determined using bicinchoninic acid assays (Thermo Fisher Scientific). Results were calculated by correcting for total protein, subtracting the uptake by cells expressing empty vector, setting WT NTCP as 100%, and then comparing all variants to WT as percent of control.

### Surface biotinylation

HEK293 cells were plated on 6-well plates and transfected as described previously. Forty-eight hours later, cells were incubated for 15 min on ice, and all solutions and buffers used were prechilled. Each well was washed with PBS and then incubated for 1 h at 4 °C while rocking with 1 mg/ml EZ-Link NHS-SS-Biotin (Thermo Fisher Scientific) in PBS. Cells were then washed with PBS and incubated for 20 min at 4 °C with 100 mM glycine in PBS while rocking. After washing with PBS, cells were lysed using 10 mM Tris, 150 mM NaCl, 1 mM EDTA, 0.1% SDS, 1% Triton X-100, pH 7.5 (lysis buffer), containing protease inhibitors (cOmplete protease inhibitor cocktail; Sigma-Aldrich). The lysates were centrifuged at 10,000*g* for 2 min, and the supernatant was incubated for 1 h at room temperature using end-over-end rotation with prewashed NeutrAvidin Agarose Resin beads (Thermo Fisher Scientific). The beads were washed with lysis buffer, captured proteins were eluted with 1× SDS sample buffer containing 5% β-mercaptoethanol and 1× protease inhibitors at 70 °C for 10 min and collected by centrifugation at 850*g* for 5 min.

### Western blotting

Surface biotinylation samples were heated to 50 °C for 10 min before separation using 4% to 20% polyacrylamide gradient gels (Bio-Rad, Hercules, CA, USA). After separation, proteins were transferred to nitrocellulose membranes using Invitrogen's Power Blotter System. Blots were blocked with 5% milk in Tris-buffered saline containing 0.1% Tween 20 for 1 h at room temperature while rocking. After blocking, blots were incubated overnight at 4 °C with a mouse antibody against the α subunit of Na^+^/K^+^-ATPase (Abcam-ab7671; 1:2000) and with a mouse antibody against the His tag (Tetra·His Antibody, QIAGEN catalog no. 34670; 1:2000) in blocking solution on a rocker. The next day blots were washed with Tris-buffered saline (TBS) containing 0.1% Tween 20 and TBS before incubation with a horseradish peroxidase–conjugated goat antimouse secondary antibody at 1:10,000 (Thermo Fisher Scientific, catalog no. 31430) in 2.5% milk in TBS. After 1 h, blots were washed with TBS and incubated with SuperSignal West Pico Chemiluminescent substrate (Thermo Fisher Scientific). Blots were visualized using a LI-COR Odyssey Fc (LI-COR, Lincoln, NE, USA), and bands were quantified using their Image Studio Lite Quantification Software.

### Time dependency and kinetics experiments

Initial linear rates were determined for WT, S267F, S267W, and S267N using multiple time points between 10 s and 10 min at low and high concentrations of each substrate: TCA at 0.1 μM and 100 μM; estrone-3-sulfate at 1 μM and 200 μM; and rosuvastatin at 5 μM and 500 μM. Kinetics for WT, S267F, S267W, and S267N were determined using HEK293 cells plated on 48-well plates. Uptakes were performed using sodium-containing and sodium-free uptake buffers (NaCl was replaced by choline chloride), and after correction for total protein and surface expression, results were analyzed using GraphPad Prism 8 (GraphPad Software Inc., San Diego, CA) (Michaelis–Menten kinetics).

### Homology model for human NTCP

To model the human NTCP sequence (National Center for Biotechnology Information: NP_003040), structural models were constructed using the SWISS-MODEL automated protein modeling server (https://swissmodel.expasy.org/) (33, 34, 35). To model the inward-open conformation of NTCP, we used as a template the structure of a bacterial homolog from *N. meningitidis* (Protein Data Bank [PDB] 3ZUY; 25% sequence identity with NTCP) (22). To model the outward-open conformation of NTCP, we used as a template the structure of a bacterial homolog from *Y. frederiksenii* (PDB 4N7X; 26% sequence identity with NTCP) (24). As with the templates on which the models were based, the topology of each model comprises nine TM helices linked by short loops into core (TM 3, 4, 5, 8, 9, and 10) and panel domains (TM 2, 6, and 7), along with the substrate-binding intracellular crevice (Fig. 6). The models of NTCP lack the N-terminal sequence corresponding to TM1 of the ASBT crystal structures; to keep the helix numbering consistent with the known crystal structures, we have chosen to start the NTCP helix numbering with TM2.

### Modeling S267 variants of human NTCP

To facilitate structural exploration in response to sequence variants, we began by using the relax protocol (36, 37, 38) in the Rosetta macromolecular modeling suite (39) to generate a close structural ensemble from each of the two homology models provided by SWISS-MODEL. Each starting conformation was used to carry out 1000 independent simulations, and the top-scoring 100 output structures were retained as a representative ensemble for the (WT) inward-open or outward-open state.

To build a structural model of a given NTCP sequence variant, we used the ddG protocol in Rosetta (40, 41). With respect to our study, this protocol was used to introduce the desired amino acid substitution at position 267 and then iterated between optimization of the nearby side chains and optimization of the backbone. We applied this protocol 10 times to each of the 100 members of our (WT) structural ensemble to yield 1000 models of the desired sequence variant. To avoid potential sampling artifacts from drawing the conformation/energy from the single lowest-energy conformation sampled, we ranked all conformations for a given sequence variant on the basis for Rosetta energy and carried forward the 50th-best conformation (*i.e.* 95th percentile) as the representative.

The same process was repeated for each of the 20 amino acids to generate all possible sequence variants at this position. Although the starting models already included serine at position 267, the same protocol was nonetheless applied to introduce serine; this ensured that any structural/energetic changes were indeed because of sequence variations and not simply changes relative to the starting structure induced by the modeling protocol.

The same analysis was separately completed using the structural ensemble for the inward-open state and outward-open state.

All Rosetta calculations were carried out using git revision 0e7ed9fd3cd610f2a7c9f3bdcaba64a9b11aab0d of the developer master source code.

The two homology models originally provided by SWISS-MODEL were also used as input for energy calculations using FOLDX, version 4 (26).

### Statistical analysis

Calculations were performed using GraphPad Prism 8. Correlation was evaluated using Pearson and Spearman correlation coefficients. Significance was determined using one-way ANOVA followed by Dunnett's multiple comparisons. Results were considered significantly different at *p* < 0.05.

## Data availability

PDB coordinates for the two homology models are deposited and freely available on Mendeley (https://doi.org/10.17632/spt9jkgy2y.1), along with the single lowest-energy Rosetta model for each variant in both the inward-open and outward-open conformations. All other data are contained within the article and in the Supporting Information.

## Conflict of interest

The authors declare that they have no conflicts of interest with the contents of this article.

## Supporting information

Supporting information

NTCP Sequence Alignment File
