# Supplementary material for: A clinically relevant polymorphism in the Na+/taurocholate cotransporting polypeptide (NTCP) occurs at a rheostat position
Source: J Biol Chem. 2020 Dec 2;296:100047. doi: 10.1074/jbc.RA120.014889 (PMC7948949; doi:10.1074/jbc.RA120.014889)
Supplement: Supporting information [file mmc1.docx]

Supporting information for:

A clinically-relevant polymorphism in the Na^+^/taurocholate cotransporting polypeptide (NTCP) occurs at a rheostat position

Melissa J. Ruggiero^1^, Shipra Malhotra^2,3^, Aron W. Fenton^4^, Liskin Swint-Kruse^4^, John Karanicolas^2^, and Bruno Hagenbuch^1,^*

^1^Department of Pharmacology, Toxicology and Therapeutics

The University of Kansas Medical Center

Kansas City, KS 66160

^2^Program in Molecular Therapeutics

Fox Chase Cancer Center

333 Cottman Avenue, Philadelphia, PA 19111

^3^Center for Computational Biology

University of Kansas

Lawrence, KS 66045

^4^Department of Biochemistry and Molecular Biology

The University of Kansas Medical Center

Kansas City, KS 66160

**Supporting Methods: Multiple Sequence Alignment**

A multiple sequence alignment of NTCP orthologs from a wide variety of species, including both prokaryotes and eukaryotes, was created using the PSI-BLAST (Basic Local Alignment Search Tool) algorithm (1) from the National Center for Biotechnology Information. Eight separate searches were completed in July 2017. The first six searches were completed using the human SLC10A family members (2) as query sequences: NTCP (*SLC10A1*), ASBT (*SLC10A2*), P3 protein (*SLC10A3*), sodium/bile acid cotransporter 4 (*SLC10A4*), P5 (*SLC10A5*), and SOAT (*SLC10A6*). Several homologs were retrieved by two or more of the searches, which facilitated the later combination of these 6 alignments into one (below). The remaining BLAST searches were completed using two NTCP bacterial homologs, one from *Neisseria meningitidis* (pdb 3ZUY; (3)) and one from *Yersinia frederiksenii* (pdb 4N7X; (4)) as queries, each of which generated thousands of search hits with sequence identities spanning 39%-99%. Following each of the 8 BLAST searches, the resulting sequences were aligned using Clustal Omega (5).

Next, to relate the eukaryotic and prokaryotic sequence alignments, the structural model built for human NTCP (see main text Methods) was super-imposed onto the 4N7X crystal structure of the bacterial homolog using UCSF Chimera (6). A structure-based sequence alignment was created from these reference proteins. This alignment was used in combination with PROMALS3D (7) to align the human SLC10A family members to the crystal structure. Finally, using sequences that were present in one or more alignments as guide sequences, the algorithm MARS (8) was used to combine the eukaryotic and prokaryotic sequences in one file without perturbing the structure-based alignments.

During these processes, sequences were curated by removing (i) duplicate sequences, (ii) particularly long or short sequences (as compared to human NTCP), (iii) sequences with large deletions, or (iv) those with ambivalent amino acid assignments. To prevent over-representation and facilitate proper sampling for subsequent calculations, bacterial sequences were clustering by their sequence identities; clear clusters were evident at the ~80% thresholds. Each of these clusters was randomly sampled to select no more than five sequences for inclusion in the final alignment of 1561 sequences (see sequence alignment file in the Supporting Information). Sequence entropies for the combined, sampled alignment was calculated using BioEdit v 7.0.5.3 software (9).

The final sequence alignment, in clustal format, is included as a text file in a separate Supplementary File.

**Supporting Table 1**: Statistical analysis of substrate uptake by wild-type NTCP and S267 variants using multiple comparisons

| **S267 variants** | **Adjusted P values Taurocholate transport** | **Adjusted P values Taurocholate transport** | **Adjusted P values Taurocholate transport** |
| --- | --- | --- | --- |
|  |  |  |  |
| WT vs. A | 0.9878 | 0.1837 | 0.0635 |
| WT vs. C | **<0.0001** | **<0.0001** | **0.0001** |
| WT vs. D | 0.0796 | 0.9999 | 0.9996 |
| WT vs. E | **<0.0001** | **0.0003** | **<0.0001** |
| WT vs. F | **<0.0001** | **0.0002** | **<0.0001** |
| WT vs. G | 0.1190 | 0.1423 | **0.0141** |
| WT vs. H | 0.9997 | 0.0899 | **<0.0001** |
| WT vs. I | **0.0016** | **0.0110** | **<0.0001** |
| WT vs. K | **<0.0001** | **0.0008** | **0.0002** |
| WT vs. L | **<0.0001** | **0.0177** | **0.0323** |
| WT vs. M | **0.0001** | **0.0307** | **<0.0001** |
| WT vs. N | **<0.0001** | 0.9993 | 0.9997 |
| WT vs. P | **<0.0001** | **<0.0001** | **<0.0001** |
| WT vs. Q | 0.2623 | 0.3553 | 0.0983 |
| WT vs. R | **<0.0001** | **0.0003** | **<0.0001** |
| WT vs. T | 0.9997 | 0.1270 | **0.0004** |
| WT vs. V | 0.9996 | 0.1475 | **0.0009** |
| WT vs. W | **0.0005** | **<0.0001** | **<0.0001** |
| WT vs. Y | **0.0047** | **<0.0001** | **<0.0001** |

Adjusted P values for each comparison were calculated using Dunnett's multiple comparisons test in GraphPad Prism version 8. Significance was set at 0.05 and significant differences are indicated in bold.

**Supporting Table 2**: Statistical analysis of initial substrate uptake normalized for surface expression multiple comparisons

| **S267 variants** | **Adjusted P values Taurocholate transport** | **Adjusted P values Taurocholate transport** | **Adjusted P values Taurocholate transport** |
| --- | --- | --- | --- |
|  |  |  |  |
| WT vs. A | 0.9993 | 0.4934 | 0.1990 |
| WT vs. C | **0.0062** | **<0.0001** | **<0.0001** |
| WT vs. D | **0.0284** | 0.9991 | 0.9943 |
| WT vs. E | 0.1533 | 0.0744 | **0.0493** |
| WT vs. F | **0.0002** | **<0.0001** | **<0.0001** |
| WT vs. G | 0.7724 | 0.2669 | **0.0337** |
| WT vs. H | 0.9832 | 0.9949 | **0.0002** |
| WT vs. I | 0.7712 | 0.4995 | **0.0011** |
| WT vs. K | **0.0021** | 0.1286 | 0.0798 |
| WT vs. L | **0.0006** | 0.1014 | 0.1104 |
| WT vs. M | **0.0017** | 0.0725 | **0.0004** |
| WT vs. N | **0.0004** | 0.9996 | 0.9998 |
| WT vs. P | **<0.0001** | **0.0030** | **0.0002** |
| WT vs. Q | **<0.0001** | **<0.0001** | **<0.0001** |
| WT vs. R | **0.0001** | **0.0128** | **0.0028** |
| WT vs. T | 0.9879 | 0.1712 | **0.0015** |
| WT vs. V | 0.9999 | 0.4063 | **0.0070** |
| WT vs. W | **0.0008** | 0.3074 | **0.0006** |
| WT vs. Y | **0.0054** | **0.0150** | **<0.0001** |

Adjusted P values for each comparison were calculated using Dunnett's multiple comparisons test in GraphPad Prism version 8. Significance was set at 0.05 and significant differences are indicated in bold.

**Supporting Table 3**: Pearson and Spearman coefficients calculated for various correlation studies

|  | Pearson | | Spearman | |
| --- | --- | --- | --- | --- |
|  | Corr. coeff. | p-value | Corr. coeff. | p-value |
| Taurocholate v Estrone-3-Sulfate (excluding outliers) | 0.9321 | **<0.0001** | 0.8265 | **0.0002** |
| Taurocholate v Rosuvastatin (excluding outliers) | 0.9143 | **<0.0001** | 0.7000 | **0.0048** |
| Estrone-3-Sulfate v Rosuvastatin (excluding outliers) | 0.9509 | **<0.0001** | 0.8702 | **<0.0001** |
| Taurocholate v Rosetta Inward | 0.0537 | 0.8273 | 0.1561 | 0.5233 |
| Estrone-3-Sulfate v Rosetta Inward | -0.1361 | 0.5785 | -0.1193 | 0.6266 |
| Rosuvastatin v Rosetta Inward | -0.1510 | 0.5372 | 0.0754 | 0.7589 |
| Taurocholate v Rosetta Outward | -0.1091 | 0.6565 | 0.2825 | 0.2413 |
| Estrone-3-Sulfate v Rosetta Outward | 0.0652 | 0.7908 | 0.1789 | 0.4636 |
| Rosuvastatin v Rosetta Outward | 0.0662 | 0.7877 | 0.2491 | 0.3037 |
| Taurocholate v Rosetta Inward Minus Outward | 0.1532 | 0.5313 | -0.0737 | 0.7643 |
| Estrone-3-Sulfate v Rosetta Inward Minus Outward | -0.2095 | 0.3892 | -0.3018 | 0.2093 |
| Rosuvastatin v Rosetta Inward Minus Outward | -0.2273 | 0.3494 | -0.1737 | 0.4770 |
| Surface Expression v Rosetta Inward | -0.3237 | 0.1765 | -0.2466 | 0.3088 |
| Surface Expression v Rosetta Outward | 0.3183 | 0.1842 | 0.3010 | 0.2105 |
| Surface Expression v Rosetta Inward Minus Outward | -0.6364 | **0.0034** | -0.5186 | **0.0229** |
| Surface Expression v FoldX Inward Minus Outward | -0.4446 | 0.0968 | -0.3861 | 0.1551 |
| Rosetta (Inward-Outward) v FoldX (Inward-Outward) | 0.5787 | **0.0238** | 0.5714 | **0.0286** |

Correlation scores from comparison figures (Figures 4, 8, and Supporting Figures 3, 4, 5) were calculated in GraphPad Prism 8 using either the Pearson correlation or the nonparametric Spearman correlation. Significance was set at 0.05 and significant differences are indicated in bold.

A

**
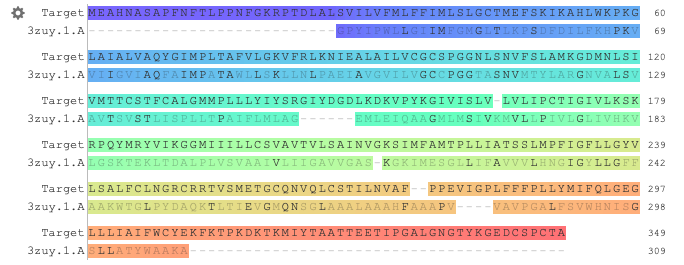
**

B

**
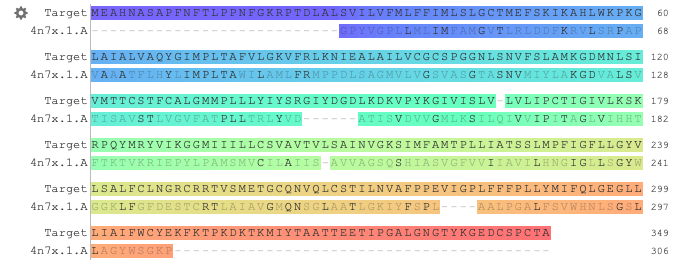
**

**Supporting Figure 1.** Pairwise sequence alignments of human NTCP against the templates used for comparative modeling. ***Top:*** Alignment to *Yersinia frederiksenii* ASBT (ASBT_Yf_, 26% sequence identity to NTCP, PDB ID 3zuy), used to model the outward-open conformation of human NTCP. ***Bottom:*** Alignment to *Neisseria meningitidis* ASBT (ASBT_Nm_, 25% sequence identity to NTCP, PDB ID 4n7x), used to model the inward-open conformation of human NTCP.

**
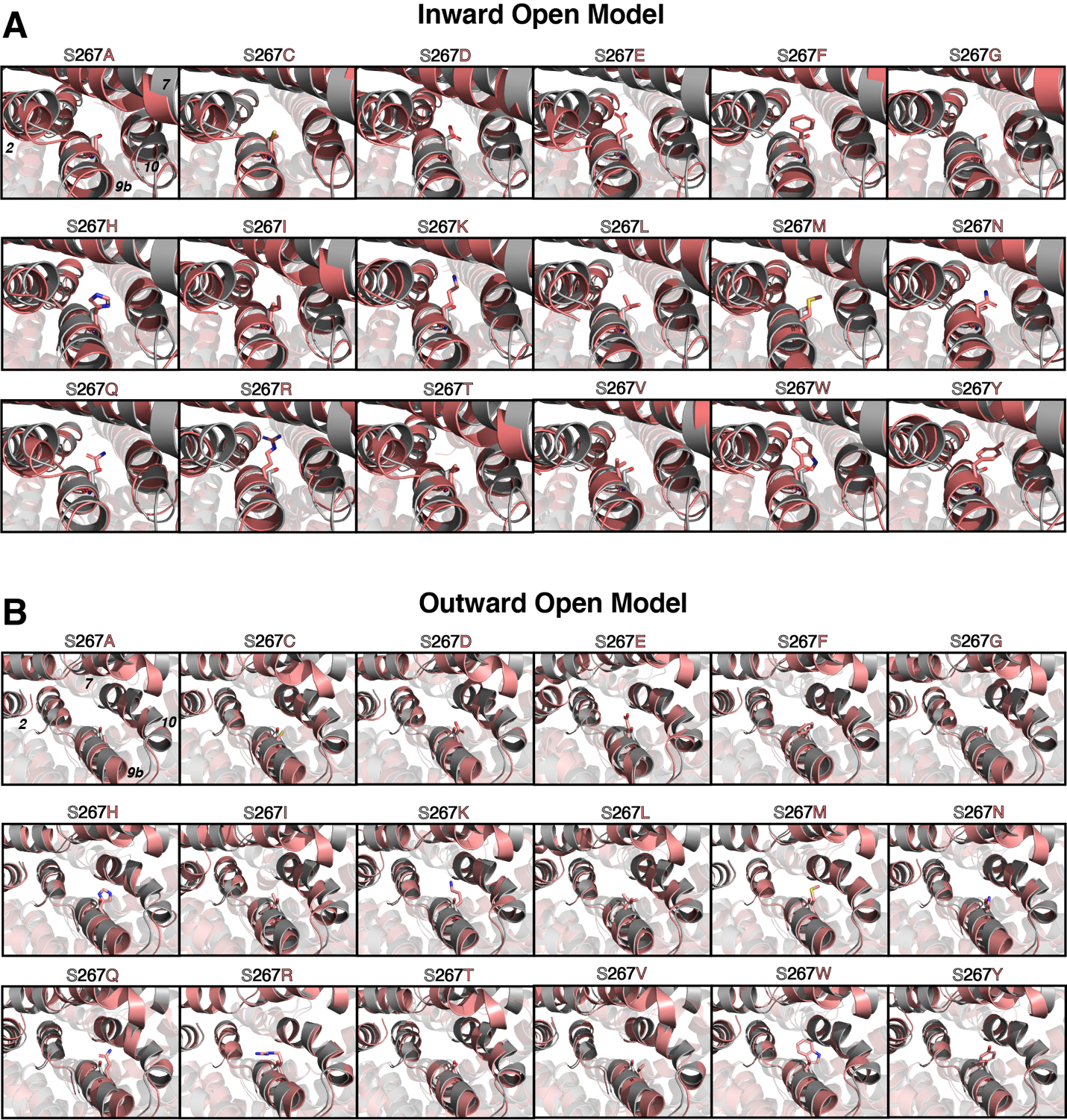
**

**Supporting Figure 2.** Sequence variation at S267 position leads to alternative local packing. Structural details are shown from WT in gray and 18 mutant structures in salmon color (proline was excluded) at the 267 position. **A.** Models in the inward-open conformation. **B.** Models in the outward-open conformation.

B

A

Supporting Figure 3. Correlation of Rosetta energies and quantification of surface expression levels. Energies calculated using Rosetta are plotted against the percent surface expression (Figure 2B) of each variant. Individual points are labeled with letters to indicate their amino acid replacements; wild-type is indicated as “WT”. A. Energy for the inward-open model, B. energy for the outward-open model.

Supporting Figure 4. Comparison of Rosetta and FoldX calculations. For each mutation, the energy differences between the Inward open and Outward open models are plotted, as calculated by Rosetta and FoldX. The FoldX values are centered at zero because all energy differences are calculated relative to the WT model for that conformation (*i.e.* there is no background energy difference between the Inward open and Outward open starting points, since both are set to zero). By contrast, Rosetta assigns a value of -12.8 to WT because it scores the Inward open conformation more favorably than the Outward open. Calculated energies are correlated with one another (p<0.03). Points are excluded for proline (Rosetta did not yield reasonable energies) and for aromatic amino acids (FoldX did not yield reasonable energies).

**Supporting Figure 5.** Correlation of Rosetta energy scores and normalized initial substrate uptake. Inward-open model **A.**, outward-open model **B.** and inward-open minus outward-open model **C.** energy scores calculated using the Rosetta software suite are plotted against normalized substrate uptake values from Figure 3. No correlations were observed.

**Supporting References**

1. Altschul, S. F., Madden, T. L., Schaffer, A. A., Zhang, J., Zhang, Z., Miller, W., and Lipman, D. J. (1997) Gapped BLAST and PSI-BLAST: a new generation of protein database search programs. *Nucleic Acids Res* **25**, 3389-3402

2. Claro da Silva, T., Polli, J. E., and Swaan, P. W. (2013) The solute carrier family 10 (SLC10): beyond bile acid transport. *Mol Aspects Med* **34**, 252-269

3. Hu, N. J., Iwata, S., Cameron, A. D., and Drew, D. (2011) Crystal structure of a bacterial homologue of the bile acid sodium symporter ASBT. *Nature* **478**, 408-411

4. Zhou, X., Levin, E. J., Pan, Y., McCoy, J. G., Sharma, R., Kloss, B., Bruni, R., Quick, M., and Zhou, M. (2014) Structural basis of the alternating-access mechanism in a bile acid transporter. *Nature* **505**, 569-573

5. Madeira, F., Park, Y. M., Lee, J., Buso, N., Gur, T., Madhusoodanan, N., Basutkar, P., Tivey, A. R. N., Potter, S. C., Finn, R. D., and Lopez, R. (2019) The EMBL-EBI search and sequence analysis tools APIs in 2019. *Nucleic Acids Res* **47**, W636-W641

6. Pettersen, E. F., Goddard, T. D., Huang, C. C., Couch, G. S., Greenblatt, D. M., Meng, E. C., and Ferrin, T. E. (2004) UCSF Chimera--a visualization system for exploratory research and analysis. *J Comput Chem* **25**, 1605-1612

7. Pei, J., Kim, B. H., and Grishin, N. V. (2008) PROMALS3D: a tool for multiple protein sequence and structure alignments. *Nucleic Acids Res* **36**, 2295-2300

8. Parente, D. J., Ray, J. C., and Swint-Kruse, L. (2015) Amino acid positions subject to multiple coevolutionary constraints can be robustly identified by their eigenvector network centrality scores. *Proteins* **83**, 2293-2306

9. Tippmann, H. F. (2004) Analysis for free: comparing programs for sequence analysis. *Brief Bioinform* **5**, 82-87
